# Inference Engine in an Intelligent Ship Course-Keeping System

**DOI:** 10.1155/2017/2561383

**Published:** 2017-11-29

**Authors:** Piotr Borkowski

**Affiliations:** Maritime University of Szczecin, Wały Chrobrego 1, 70500 Szczecin, Poland

## Abstract

The article presents an original design of an expert system, whose function is to automatically stabilize ship's course. The focus is put on the inference engine, a mechanism that consists of two functional components. One is responsible for the construction of state space regions, implemented on the basis of properly processed signals recorded by sensors from the input and output of an object. The other component is responsible for generating a control decision based on the knowledge obtained in the first module. The computing experiments described herein prove the effective and correct operation of the proposed system.

## 1. Introduction

The importance of maritime transport in the global economy is expressed primarily by the quantity of cargoes carried by sea, currently estimated at 80%. If we assume the transport performance as a criterion, the share of maritime transport in global trade exceeds 90% [1]. The actual competitiveness of maritime transport in relation to other modes results in increasingly larger seaborne trade. Consequently, traffic intensity, tonnage, and speeds of vessels also increase. This in turn affects the level of safety of people, vessels, cargo, and the environment. In order to improve safety and increase the efficiency and competitiveness of marine transport services, more and more advanced navigational systems and equipment are installed on board ships and in land-based vessel traffic control centers. The benefits are as follows:

- Material benefits associated with the reduction of losses and damage to and sinking of vessels
- Economic benefits due to lower operating costs and shorter voyage
- Environmental protection and prevention of environmental disasters caused by collisions of vessels carrying dangerous goods

One of the typical tasks related to the safety of navigation is the problem of automatic course stabilization, consisting in automatic maintenance of the ship on a preset course under conditions of external disturbances, as well as bringing the ship automatically back on a preset course after its previous alteration. The ship's course-keeping capability is crucial, because nonoptimal control of the rudder involves the loss of average speed, covering a longer track, lengthened time of the voyage, and more fuel consumption, which in consequence raises total operating costs. The associated problem is steering gear overload that may lead to a critical breakdown. Most importantly, uncontrollable yawing, especially on waterways with increased traffic, affects the level of safety, increasing a risk of collision [2].

The history of automatic ship movement control devices dated back to the early 20th century. The invention of gyrocompass (1908, H. Anschutz), a device capable of indicating north with high accuracy, also on ships made of steel (unlike the compass), enabled the development of autopilots (1911, E. Sperry). Initially, such devices were simple mechanisms, transforming proportionally a signal from the gyrocompass into a control decision, so their designs were mostly based on the concept of the P regulator. From this period the publications [3, 4] are worth noting, regarded as pioneer works in the field.

The helmsman's actions on the bridge are similar to the operation of PID controller, that is, it proportionally accounts for deviation from ship's course (term P: a proportional component), action of factors causing permanent deviation from the course (term I: an integral component), and speed of deviating from the course (term D: a derivative component). It is therefore natural that designs of subsequent autopilots usually were based on the PID controller concept, sometimes on PD controllers. Till the 1990s, the PID control method was a dominant solution, and specific examples can be found in the works [5–7].

Because dynamic properties of a ship are not constant (they depend on vessel type and sailing conditions), the PID/PD controller setpoints should also change, especially if the autopilot is to serve for maintaining the ship on course, as well as for course alteration to a new value. This requirement has initiated a whole range of new concepts based on the notion of space states and stochastic control theory. These solutions often make use of Kalman filter or reference model methods. Examples of adaptive autopilot designs can be found in works [8–15].

The simplest adaptive autopilots based on the PID (PD) controller concept have higher quality of control than previous solutions. However, their idea is based mainly on the linear model of object dynamics. Developments of the control theory included attempts to design autopilots operating on the basis of nonlinear control techniques. Some examples can be found in the publications [16–25].

Another solution bypassing the problem of nonlinearity is the use of computer technologies and artificial intelligence methods. The number of publications on this subject is constantly on the rise. Example references are [26–36]. The system herein described fits into that area of research. It is not a classic expert system, where expert knowledge is recorded in the form of rules. In the proposed solution, the knowledge is represented by properly processed signals recorded by sensors from the object input and output. The inference engine is crucial for the proposed expert system of ship's course stabilization. It consists of two functional components or modules. One of them is responsible for the construction of space states, a basis for the other module to generate a control decision.

In this original method, it is possible to overcome difficulties that occur in developing typical control algorithms for a complex and nonlinear model affected by strong external disturbances (wind, waves), while providing high quality control.

## 2. The Design of an Intelligent System of Ship Course Stabilization

**Figure 1:**
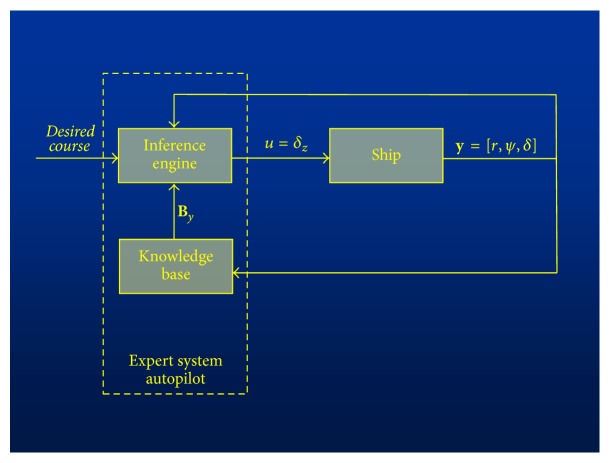
The circulation of signals in the proposed expert system.

The circulation of signals in the proposed expert system of ship course stabilization (course autopilot) is shown in Figure 1. In the inference engine, for a given object output **y** = [*r*, *ψ*, *δ*] (*r*: angular velocity, *ψ*: deviation from course, and *δ*: rudder angle), a control decision *u* is generated, that is, the preset rudder angle *δ*_*z*_, on the basis of quantities retrieved from the knowledge base **B**_*y*_. It is so determined as to stabilize the defined quantity, that is, ship's course. This process is repeated at time unit intervals Δ*t*.

Let the symmetric, closed interval 〈*r*_min_, *r*_max_〉 be a set of all angular velocities that the object may assume. After discretization, we obtain this set:

(1) rmin,rmin+Δr,rmin+2Δr,…,rmin+nr−1Δr=rmax,

where

 
Δ*r* is discretization unit,
 
*n*_*r*_ is number of elements in the set (1).

Similarly, as a result of discretization of possible values of rudder angle (i.e., the interval 〈*δ*_min_, *δ*_max_〉), we get a set:

(2) δmin,δmin+Δδ,δmin+2Δδ,…,δmin+nδ−1Δδ=δmax,

where

 
Δ*δ* is discretization unit,
 
*n*_*δ*_ is number of elements in the set (2).

The knowledge base contains object output signals recorded in an ordered manner (resp., angular velocity, deviation from the course, and rudder angle), depending on the discretized state of the object and discretized control decisions. The output signal is here to be understood as measurement of object state after one time unit Δ*t*. For practical reasons, the signals may be replaced by data obtained from any complex hydrodynamic model describing the object and the impact of the environment. However, to avoid a system with an open loop, a hydrodynamic model has to be adapted by a parametric or structural method to changing conditions, on the basis of real signals recorded from the object output. An ordered record of signals may be implemented in the form of multidimensional arrays with these values:

(3) $$\begin{array}{c} r\left[\;i,j,k\;\right];\\ \psi \left[\;i,j,k\;\right];\\ \delta \left[\;i,j\;\right]\\ \;\left(0\leq i<n_{\delta };\;\;0\leq j<n_{\delta };\;\;0\leq k<n_{r}\right), \end{array}$$

where

 
*r*[*i*, *j*, *k*] is value of object angular velocity after one time unit Δ*t*; when at a current instant the angular velocity of the object is *r*_min_ + *k*Δ*r*, rudder angle is *δ*_min_ + *j*Δ*δ*, and the control decision has the form *δ*_min_ + *i*Δ*δ*,
 
*ψ*[*i*, *j*, *k*] is value of object course deviation after one time unit Δ*t*; when at a current instant the object angular velocity is *r*_min_ + *k*Δ*r*, rudder angle is *δ*_min_ + *j*Δ*δ*, course deviation is equal to zero, and the control decision has the form *δ*_min_ + *i*Δ*δ*,
 
*δ*[*i*, *j*] is value of rudder angle after one time unit Δ*t*; when at a current instant the rudder angle is *δ*_min_ + *j*Δ*δ*, and the control decision has the form *δ*_min_ + *i*Δ*δ*.

Array values (3) and linear interpolation used allow determining an object output for any initial state and any control decision in the form as elements of the set (2).

The following sections describe the inference engine of the proposed expert system, the crucial component in generating a control decision. The last section provides a proof of control system stability.

### 2.1. The Construction of State Space Regions

The first of two functionally important components of the inference engine of the proposed expert system is responsible for the construction of state space regions. A proper classification of state space regions will be a basis for the control algorithm described in the next section.

Let the closed zero region *O*_0_ be a set of object states in the form:

(4) $$\begin{array}{c} O_{0}=\left\{\;\left(\;r,\psi ,\delta \;\right):\left|\;r\;\right|\leq r_{0}\wedge \left|\;\psi \;\right|\leq \psi_{0}\wedge \left|\;\delta \;\right|\leq \delta_{0}\;\right\}, \end{array}$$

where

 
*r*_0_, *ψ*_0_, *δ*_0_ is close to zero, positive values determined arbitrarily (*r*_0_∈ (1), *δ*_0_∈ (2)).

The zero region *O*_0_ is therefore a cuboid with the boundary. Parameters *r*_0_, *ψ*_0_, and *δ*_0_ should be arbitrarily selected so that their values are close to zero. They are thus established because the problem of ship course stabilization consists in bringing simultaneously the values of the angular velocity, course deviation, and rudder angle close to zero.

**Figure 2:**
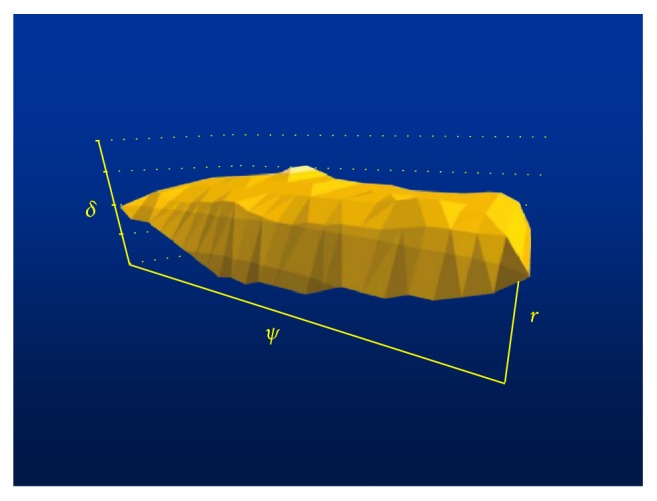
Visualization *O*_*k*_.

The closed *k*th region *O*_*k*_ (Figure 2) where *k* ∈ *N*_+_ is a set of object states for which there exists a sequence of control decisions not longer than *k*, generated at time unit intervals, bringing the object state to zero region *O*_0_.

The first region *O*_1_ will be a set of such object states for which there exists a control decision that when executed results in the object state after one time unit Δ*t* getting to the zero region *O*_0_. The second region *O*_2_ is the sum of the first region *O*_1_ and a set of object states for which there exists a control decision, leading to the state transition to the first region *O*_1_ after one time unit Δ*t*. In the same way, further regions are determined. It should be noted that the so-defined process of determining regions of state spaces is finite, because a set of all possible states is a cuboid with the boundary (angular velocity, deviation from the course, or rudder angle may only take values from a certain range). So there is some *k*_max_ ∈ *N*_+_, such that the region with this index will be a set of all possible states of the object (space of states):

(5) $$\begin{array}{c} O_{k_{max}}=\langle \;r_{min},r_{max}\;\rangle \times \langle \;-\pi ,\pi \;\rangle \times \langle \;\delta_{min},\delta_{max}\;\rangle . \end{array}$$

The boundaries of state space regions are determined ascendingly starting from the first region *O*_1_, to the region *O*_*k*_max__. It can be noticed that for preset angular velocity and rudder angle, based on the values from the arrays (3), we can determine the ranges of course deviations for which there exist a control decision that after execution will result, after one time unit Δ*t*, in the object state being moved to the region with an index lower by one than the region under consideration. With that relationship used iteratively, we get the boundaries of state space regions determined in a discrete manner. They can be written as two multidimensional arrays with the values:

(6) $$\begin{array}{c} o\min\left[\;i,j,k\;\right];\\ o\max\left[\;i,j,k\;\right]\\ \;\left(0\leq i<n_{r};\;\;0\leq j<n_{\delta };\;\;0\leq k<k_{max}\right), \end{array}$$

where

 
*o*min⁡[*i*, *j*, *k*] is value of the lower range limit of course deviation of the* k*th region *O*_*k*_ for object angular velocity *r*_min_ + *i*Δ*r* and rudder angle *δ*_min_ + *j*Δ*δ*,
 
*o*max⁡[*i*, *j*, *k*] is value of the upper range limit of course deviation of the* k*th region *O*_*k*_ for object angular velocity *r*_min_ + *i*Δ*r* and rudder angle *δ*_min_ + *j*Δ*δ*.

The said course deviation range is to be understood as

(7) ∅while  omin⁡i,j,k=omax⁡i,j,k,omin⁡i,j,k,omax⁡i,j,kwhile  omin⁡i,j,k<omax⁡i,j,k,−π,omax⁡i,j,k∪omin⁡i,j,k,πwhile  omin⁡i,j,k>omax⁡i,j,k.

The determination of state space region boundaries (6) should take place for each change in the array values (3). Such procedure assures the adaptation of the system under consideration to changing conditions of ship movement.

### 2.2. The Control Algorithm

For the object output **y** = [*r*(*t*), *ψ*(*t*), *δ*(*t*)] at instant *t*, object states after a time unit [*r*(*t* + Δ*t*), *ψ*(*t* + Δ*t*), *δ*(*t* + Δ*t*)] are determined by means of elements retrieved from the knowledge base (**B**_*y*_: *r*[0 : *n*_*δ*_ − 1, *j* : *j* + 1, *k* : *k* + 1], *ψ*[0 : *n*_*δ*_ − 1, *j* : *j* + 1, *k* : *k* + 1], *δ*[0 : *n*_*δ*_ − 1, *j* : *j* + 1], where *r*_min_ + *k*Δ*r* ≤ *r*(*t*) < *r*_min_ + (*k* + 1)Δ*r*, *δ*_min_ + *j*Δ*δ* ≤ *δ*(*t*) < *δ*_min_ + (*j* + 1)Δ*δ*), depending on the discrete control decisions. It should be noted that for sufficiently small time increments Δ*t* the system will respond differently for some, not all, control decisions. This will limit the set of further analyzed cases.

The next step, made on the basis of array values (6), and with use of linear interpolation, defines the minimum indexes of regions where the object states will be found in the first step, depending on the analyzed control decisions (those, for which the system response was different after one time unit Δ*t*).

The generated control decision should correspond exactly with the smallest index determined in the previous step. However, this condition may prove to be inadequate, because it will be met by more than one of the analyzed control decisions. Let the set Δ_*z*_ mean all such control decisions. In this case, the most appropriate seems to be the decision that fulfils the condition:

(8) $$\begin{array}{c} \delta_{z}=\underset{\delta \in \Delta_{z}}{\min}\left\{\;\left|\;\delta \left(\;t\;\right)-\delta \;\right|\;\right\}. \end{array}$$

Condition (8), on the one hand, ensures uniqueness of the algorithm; on the other hand, it minimizes the load of the steering gear, because it minimizes the absolute difference between the current rudder angle and the control decision being generated.

The control algorithm can therefore be described in three steps:

1. Determination of the future object states depending on the discretized control decisions
2. Determining possible regions for an object state
3. Determination of the control decision satisfying condition (8)

All the steps in the algorithm are sequential. The convergence of the algorithm is guaranteed by the feasibility of each step.

The idea behind the proposed control process is that at successive instants the generated control decisions will result in the object state getting to a region with a lower index. It follows that when at the instant zero the object state will be in the *k*th region, such a control decision will be generated that at 1st instant the state object was in *k* − 1st region, until finally in *k*th instant the object state should be found in the zero region and remain there, which is equivalent to bringing the ship's course to the set value (9).

 The idea behind the proposed control process is as follows:

(9) Okt=0⟶Ok−1t=1⟶Ok−2t=2⟶⋯⟶O1t=k−1⟶O0t=k⟶O0t=k+1.

The automatic control algorithm is required to guarantee the stability of the system. The relevant proof concerning the presented method of control is given in the next section.

### 2.3. The Control System Stability

The control system is called stable (in the Lyapunov sense) then and only then, if for every positive number *ε* we can select such a number *η* (generally dependent on *ε*) that a trajectory starting in a state **x**(0) lying inside the sphere with radius *η* will remain inside the sphere with radius *ε* for any instant *t*. In addition, if the condition occurs

(10) $$\begin{array}{c} \underset{t\to \infty }{lim}x\left(\;t\;\right)=0 \end{array}$$

(i.e., for a time *t* tending to infinity the control system returns to the state of equilibrium that had been disturbed), the control system is asymptotically stable. The stability of the control system near the point of equilibrium is called local stability, and the stability at any large initial conditions **x**(0) is called the global stability.

In order to show the global stability of the control system under consideration, let for any *ε* meeting the condition (2 ≤ *i* ≤ *k*_max_, due to the definition of zero region, *d*_max_(1) is any small value, which can be considered approximately as zero)

(11) $$\begin{array}{c} d_{max}\left(\;i-1\;\right)<\varepsilon \leq d_{max}\left(\;i\;\right), \end{array}$$

where

 
*d*_max_(*i*) is the maximum distance (in the sense of Euclidean metric) of the boundary of *i*th region from the point of equilibrium of the control system (0 ≤ *i* ≤ *k*_max_).

*η* will be selected as follows:

(12) $$\begin{array}{c} \eta =d_{\min}\left(\;i-1\;\right), \end{array}$$

where

 
*d*_min_(*i*) is the minimum distance (in the sense of Euclidean metric) of the boundary of *i*th region from the point of equilibrium of the control system (0 ≤ *i* ≤ *k*_max_).

Then the initial state of the control system, lying inside the sphere with radius *η*, must remain inside the sphere with radius *ε*, because in accordance with the control algorithm discussed in the previous section, it cannot leave the region it was previously found in, which completes the proof of stability in the Lyapunov sense of the proposed control system.

The operating principle of the control process described in the previous section was that in the subsequent instants such control decisions are generated that, when out of equilibrium, the system returns to the zero region, that is, the point of equilibrium, which fulfils condition (10), proving the asymptotic stability of the control system.

## 3. Computing Experiments

The experiments have been made in the MATLAB/Simulink environment. A de Wit-Oppe model [37] was used as an object (vessel), incorporating the dynamics of the steering gear [5]:

(13) x˙1=x5cos⁡x3−x6sin⁡x3,x˙2=x5sin⁡x3+x6cos⁡x3,x˙3=x4,x˙4=−a1x4−a2x43+a3u,x˙5=−fx5−Wx42+S,x6=−r1x4−r3x43,u˙=uz−u,uz≤umax,u˙≤u˙max,

where

 
(*x*_1_, *x*_2_) = (*x*, *y*) is Cartesian coordinates (ship's position),
 
*x*_3_ = *ψ* is deviation from the course,
 
*x*_4_ = *r* is angular velocity,
 
*x*_5_ is longitudinal speed,
 
*x*_6_ is lateral speed,
 
*u* = *δ* is rudder angle,
 
*u*_*z*_ = *δ*_*z*_ is preset rudder angle,
 
*δ*_max_ is maximum rudder angle,
 
${\dot{\delta }}_{max}$ is maximum rate of turn of the rudder,
 
*S* is propeller thrust,
 
*a*_1_, *a*_2_, *a*_3_, *f*, *W*, *r*_1_, and *r*_3_ are coefficients determined from model tests (different for different types of vessels).

The ship movement parameters assumed here are those of a ship of mariner class, such as the m.s. Compass Island [37]: *a*_1_ = 0,018 [1/s], *a*_2_ = 37,2 [s/rad^2^], *a*_3_ = 0,001 [1/s^2^], *f* = 0,014 [1/s], *W* = 124 [m/rad^2^], *S* = 0,11 [m/s^2^], *r*_1_ = −69,5 [m/rad], and *r*_3_ = 0 [m·s^2^/rad^3^]. The selected ship has the following characteristics: gross registered tons 9214 [t], 13498 DWT [t], single screw, length 172 [m], maximum draft 8 [m], maximum speed 20 [kn], maximum (minimum) angular velocity *r*_max_ = 0,0191 [rad/s] (*r*_min_ = −0,0191 [rad/s]), maximum (minimum) rudder angle *δ*_max_ = 0,6 [rad] (*δ*_min_ = −0,6 [rad]), and maximum (minimum) rate of turn of the rudder ${\dot{\delta }}_{max}=0\text{,}066\;\left[rad/s\right]$ (${\dot{\delta }}_{min}=-0\text{,}066\;\left[rad/s\right]$).

In order to take account of disturbances, simulations included a signal characteristic of wind-induced sea waves [5].

There were two computing experiments. Each of them consisted of simulation series comparing the operation of the proposed system to the control performed by an LQR controller. For comparison, an LQR regulator was chosen, as it is an optimal regulator for a linear stationary object. A Nomoto model was used as a ship's model for the synthesis of the LQR regulator [5]:

(14) x˙3=x4,x˙4=−a1x4+a3u.

As a quality criterion, this functional was taken:

(15) $$\begin{array}{c} J=\overset{t}{\underset{0}{\int }}\left(\;\psi^{2}+\lambda \delta^{2}\;\right)dt, \end{array}$$

where

 
*λ* is a coefficient greater than zero, which is interpreted as a compromise between the deviation from the course (angle of yaw) and the rudder angle (steering gear load), arbitrarily adopted here as 1.

Then the control law for LQR can be described by this formula:

(16) $$\begin{array}{c} \delta =k_{\psi }\psi +k_{r}r, \end{array}$$

where

(17) kψ=−1λ,kr=1a3a1−a12+2a3λ.

**Figure 3:**
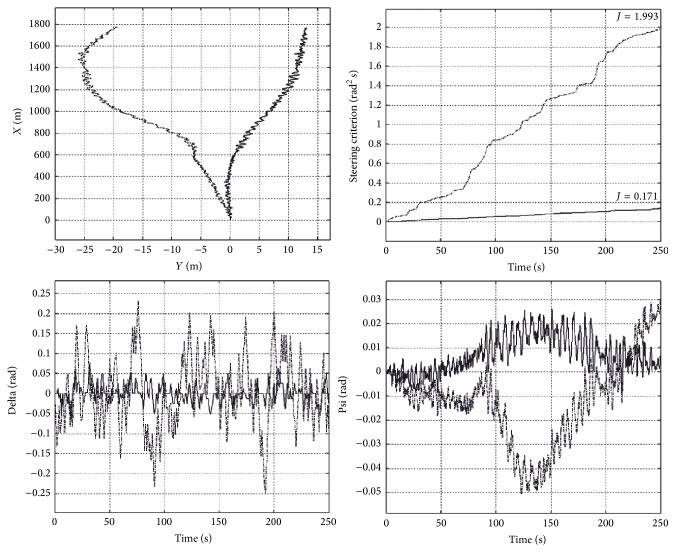
Comparison of the proposed system operation (continuous line) and an LQR controller (dashed line), experiment one.

The first computing experiment was aimed to compare the values of quality control indicator (15) measured (for a time span of 0–250 seconds) for different types of waves, for a ship with the course set on zero radians.  Figure 3 presents an example simulation result for sea state 4. The charts, respectively, depict the trajectory of ship movement, the indicator of quality control, the rudder angle, and the deviation from the course in the simulation. The values of quality control indicator should be interpreted accordingly as 0.1993% and 0.0171% decrease in ship's operational speed caused by additional resistance associated with disturbances. By using the proposed method, the decrease in speed was much smaller. These results clearly confirm high quality of the proposed system operation.

In all the cases examined, the quality indicator value for a ship controlled by the proposed system was lower than in the system using an LQR controller (even if the latter is optimal for linear stationary object). The developed method allowed reducing the decrease in ship's operational speed by 0.1%–0.5%, depending on wave height. It was also observed that the control quality was improving, compared to the LQR controller, with increasing wave height.

**Figure 4:**
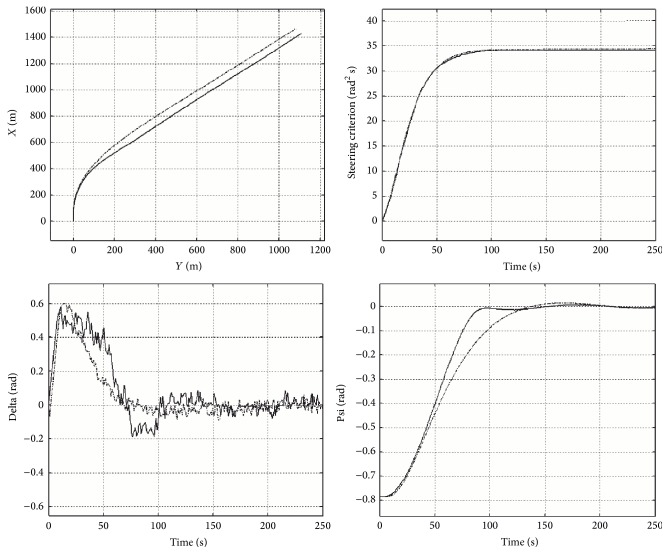
Comparison of the proposed system operation (continuous line) and an LQR controller (dashed line), experiment two.

The other computing experiment was to examine how much time it takes for the object to reach the zero region (i.e., vessel is brought on a new course), if at instant zero the course is altered from 0 radians to a new course other than zero. In other words, the test included the time of course stabilization (control time) and overshoot, in a situation of an abrupt change of the setpoint.  Figure 4 presents an example simulation result obtained during the tests. The charts illustrate the trajectory of ship movement, the indicator of quality control, the rudder angle, and the deviation from the course in the simulation. In this situation, the course was changed from 0 to *π*/4 radian. We can observe a shorter transitional period in the proposed control method. These results, like in the first experiment, confirm high quality of the proposed system.

In all the cases examined, the time to reach the zero region by a ship controlled by the proposed system was less than in case of the control involving an LQR controller. Owing to the developed method, course stabilization time was shortened approximately from 5% to 40%, depending on the value of the newly defined course.

The computing experiments presented in this section clearly confirm high quality of control (ship course stabilization) of the proposed expert system. This refers to the minimization of control criterion value (15), control time, and the magnitude of overshoot at a step alteration of the ship's course. In all the cases examined, the control quality of the proposed system was higher than that of an LQR controller.

## 4. Conclusions

One of the important tasks in maritime transport, which has an impact on the reduction of transport costs and enhancement of navigation safety, is the problem of automatic ship's course stabilization. However, the main difficulties that appear in the design of efficient navigational devices are problems with developing effective algorithms implementing desired functionalities. Designing solutions that will be sufficiently effective and based on few simplifications leads to complex systems that are hard to be analyzed in a traditional manner. Hence, it seems necessary to apply computational mathematics for solving problems of marine navigation, or, in a broader perspective, maritime transport. Computational mathematics is understood here as a combination of traditional mathematical tools, characterized by strict reasoning (strict evidence) and artificial intelligence methods, additionally supported by technical capabilities of modern computers [38]. The expert system discussed herein, tasked to automatically stabilize ship's course, fits well into that area of research. By using the data on the system hydrodynamics and the original inference engine, we could bypass difficulties that occur in developing typical control algorithms for complex and nonlinear model affected by strong external disturbances (wind, waves), while providing high quality of control. This was confirmed by computing experiments.

The proposed system will be implemented in the executive module of the navigational decision support system NAVDEC [39–41]. The executive module combined with other modules (e.g., module of data fusion [42–45] and prediction module [46, 47]) is part of the navigational decision support system in the process of safe ship conduct (invention [48]).

The developed system has a function of automatic ship's course stabilization. However, the methodology can be easily extended and used for solving problems of automatic ship steering along a preset trajectory. It seems that in the context of the proposed approach it is possible, for further considerations, to extend tasks and include other objects of control. Hence, the proposed methodology represents a new branch of intelligent control systems.
